# Sleep in neurodegenerative diseases: A focus on melatonin, melanin‐concentrating hormone and orexin

**DOI:** 10.1111/jne.70085

**Published:** 2025-08-31

**Authors:** Simon J. Guillot, Pierre‐Hervé Luppi, Luc Dupuis, Matei Bolborea

**Affiliations:** ^1^ University of Strasbourg, INSERM, UMR‐S 1329, Strasbourg Translational Neuroscience & Psychiatry Strasbourg France; ^2^ Centre of Neuroscience of Lyon CNRS/INSERM, UMR 5292/UMR 1028 Lyon France

**Keywords:** circadian rhythm, hypothalamus, neurodegenerative disease, sleep

## Abstract

Sleep and circadian rest‐activity rhythm alterations are recognised as inherent clinical features of various neurodegenerative diseases. Traditionally viewed as secondary manifestations of neurodegeneration, recent studies have revealed that disruptions in circadian rhythm and sleep–wake cycles can precede clinical symptoms and significantly contribute to the underlying pathophysiological progression. In this review, we summarise recent research on the impact of sleep and circadian rhythm alterations in ageing and major neurodegenerative diseases, including Alzheimer's, Parkinson's, Huntington's, amyotrophic lateral sclerosis, and frontotemporal dementia, highlighting the roles of melatonin, orexin, and melanin‐concentrating hormone (MCH) systems as key regulators at the intersection of sleep and neurodegeneration. We argue that sleep and circadian alterations may serve as early biomarkers and therapeutic targets for these diseases.

## 1 INTRODUCTION

Sleep is a core physiological process that is critical for maintaining optimal health and well‐being. While asleep, the body undergoes a series of restorative processes, including memory consolidation, tissue repair, and metabolic regulation.^1^ However, sleep quality and quantity are not static and change progressively with age, influenced by both physiological ageing and neurodegenerative processes.^2^, ^3^, ^4^

In the fifties, sleep has been segmented into two distinct states, one showing rapid eye movements (REM) and muscle atonia named REM sleep and the other characterised by slow oscillations named non‐rapid eye movement (NREM).^5^, ^6^, ^7^ NREM sleep is further divided into three progressive stages: NREM1, NREM2, and NREM3.^8^, ^9^ NREM1 represents the transition from wakefulness to sleep and is characterised by a low amplitude of the EEG.^10^ NREM2 follows, during which sleep deepens, body temperature drops, and the EEG shows 14 Hz oscillations named sleep spindles.^11^ Finally, NREM3 is the deepest sleep stage, characterised by delta slow wave activity. Sleep is essential for physical restoration, memory consolidation, and immune function.^9^, ^12^ In diurnal species like humans, sleep occurs at night,^13^ while it mostly occurs during the day in nocturnal species such as rodents.^14^ REM and NREM sleep alternate during the rest period, with a predominance of NREM sleep at the beginning and of REM sleep at the end.^9^, ^15^

Animal models are widely used in neurodegenerative research as surrogates for human disease. Multiple models are often employed for a single condition because no perfect model has yet been identified. The complexity of human pathologies, particularly in diseases like ALS or Alzheimer's, involves diverse genetic, cellular, and systemic factors that can only be partially modelled. Each animal model allows researchers to isolate and investigate a specific mechanism (e.g., protein aggregation, synaptic dysfunction) under controlled conditions. Furthermore, since these diseases often arise from different gene mutations leading to distinct symptom profiles, cross‐validating findings across various models helps identify therapeutic targets that are robust across multiple genetic contexts.

An interplay between circadian and homeostatic processes regulates sleep timing, propensity, and quality. Two different homeostatic processes regulate sleep depth and REM sleep duration. The homeostasis of NREM sleep is characterised by the high power of slow oscillations at the beginning of sleep, decreasing progressively until awakening. REM sleep duration is homeostatically regulated. The regulation of slow oscillations' power interacts with the circadian rhythm to generate a regenerative and robust period of sleep.^16^, ^17^, ^18^, ^19^ It regulates wake and sleep timing and duration.^20^ Complex neuronal networks are involved in the generation of the sleep–wake cycle. They include the orexin/hypocretin neurons, aminergic and cholinergic neurons for inducing wakefulness, the ventrolateral preoptic nucleus for inducing NREM sleep, and brainstem and hypothalamic neurons like the melanin‐concentrating hormone (MCH) neurons to induce REM.^21^

The circadian timing system (CTS) is driven in mammals by a central pacemaker located in the hypothalamic suprachiasmatic nuclei (SCN), which oscillates with a near‐24‐h period and is synchronised to the 24‐h day by external cues such as light, food intake, and social interactions.^22^, ^23^ The SCN regulates peripheral molecular clocks in all nucleated cells through rhythmic signals like hormone fluctuations and temperature cycles.^24^, ^25^

Each molecular clock, composed of 15 major genes operating in feedback loops, generates a circadian rhythm that coordinates feeding, fasting, anabolism (nutrient storage during wakefulness),^26^ and catabolism (glycogenolysis and lipolysis during sleep).^27^

Sleep is strongly modified across the lifespan, occupying most of the time at birth and progressively decreasing during childhood before stabilising in adults and declining again during ageing.^28^ Alterations in sleep can have far‐reaching consequences for cognitive function, emotional regulation, and physical health in older adults.^29^ Furthermore, the ageing process itself is a major risk factor for the development of neurodegenerative diseases.^30^

Neurodegenerative diseases, including Alzheimer's disease (AD), Parkinson's disease (PD), and amyotrophic lateral sclerosis (ALS), have been shown to induce sleep alterations.^31^, ^32^ The pathophysiological mechanisms underlying these pathologies have been shown to involve neural circuits regulating sleep. For instance, amyloid‐β (Aβ) plaque accumulation in Alzheimer's disease has been linked to sleep–wake cycle disruptions.^33^, ^34^ In Parkinson's disease, degeneration of dopaminergic, glutamatergic, and glycinergic neurons composing the muscle atonia generating network occurring during REM sleep is involved in REM sleep behaviour disorder (RBD) and excessive daytime sleepiness.^35^, ^36^ ALS‐related motor neuron loss can also lead to respiratory insufficiency, causing sleep‐related breathing disorders like sleep apnoea and hypoventilation.^37^, ^38^

In recent years, the bidirectional relationship between sleep and neurodegeneration has gained significant attention. Insufficient sleep quality may accelerate the progression of neurodegenerative diseases, while the progression of these diseases further alters sleep architecture.^39^, ^40^ This vicious cycle highlights the need to investigate the mechanisms underlying sleep alterations in ageing and neurodegenerative diseases and to develop targeted interventions to improve sleep quality in these populations.^41^

This review delves into the intricate interplay between sleep, ageing, and neurodegeneration, discussing age‐related sleep changes, the impact of neurodegenerative diseases on sleep architecture, and potential therapeutic approaches to mitigate sleep alterations in affected individuals. We choose here to specifically focus on melatonin, MCH, and orexin neurons, as degeneration of these neuronal populations appears frequent in most neurodegenerative diseases. This does not exclude the involvement of other sleep‐controlling neurons, particularly monoaminergic ones, which are covered in other reviews.^42^, ^43^, ^44^, ^45^, ^46^, ^47^, ^48^

## 2 SLEEP ACROSS AGEING

As individuals age, they naturally experience changes in their sleep patterns. These changes are part of the developmental and ageing processes and occur throughout their lives, with sleep duration, quality, and architecture all undergoing modifications. One aspect of ageing that affects sleep is the shift in an individual's preferred timing of sleep and wakefulness, known as chronotype.^2^ Chronotype refers to an individual's innate preference for sleeping early or late within a 24‐h cycle. It is influenced by the circadian system, which regulates sleep–wake cycles by means of internal biological clocks. Chronotype is mostly regulated by genetic factors. There are three main chronotypes: morning, intermediate, and evening types.^49^ As we age, our chronotype gradually shifts towards a morning type, leading to noticeable changes in sleep timing and architecture.^50^, ^51^ Age‐related changes within the homeostatic sleep‐regulating system can impact sleep timing, quantity, and quality. Indeed, the discrepancy between circadian and homeostatic processes results in sleep asynchrony during ageing.^52^, ^53^ Further, multiple endocrine and neuroendocrine adjustments accompany chronotype shifts.^54^, ^55^, ^56^, ^57^ Melatonin, serotonin, and cortisol are three key regulators of circadian rhythm. Melatonin, secreted by the pineal gland at dark onset, shows a gradual decline during ageing due to pineal gland calcification.^58^, ^59^ Conversely, elevated levels of cortisol^60^, ^61^ and brain‐derived serotonin correlate with poorer ageing trajectories,^62^, ^63^, ^64^ which are themselves associated with decreased sleep efficiency and increased sleep fragmentation.^65^, ^66^ Interestingly, it has been shown that the function of MCH and orexin neurons is altered as we age.^67^, ^68^, ^69^, ^70^, ^71^, ^72^, ^73^, ^74^, ^75^, ^76^, ^77^, ^78^, ^79^ For example, orexin levels have been observed to rise in older individuals, and orexin neurons become progressively hyperexcitable,^79^ potentially contributing to insomnia and sleep–wake disruptions.^77^, ^80^, ^81^ However, the number of orexin neurons in the lateral hypothalamic area (LHA) decreases with age.^74^, ^79^ The fate of MCH‐positive neurons across the ageing *continuum* remains unknown. As neuronal networks gradually deteriorate, sleep macroarchitecture, including NREM and REM cycles, undergoes progressive modifications, resulting in fragmented sleep.^82^ Individuals across various life stages may encounter challenges in falling asleep, reduced time spent in NREM2, NREM3, and REM sleep, and increased nighttime wakefulness. These sleep alterations can negatively impact cognitive function, quality of life, and neurodevelopmental trajectories.^83^, ^84^ Recent investigations more specifically demonstrate that age‐related changes significantly influence sleep oscillations at the microarchitectural level, affecting their density and duration.^85^, ^86^ A decline in homeostatic sleep pressure leads to reorganisation of NREM3, marked by a reduction in slow oscillations' amplitude and density.^85^ Sleep spindles, slow oscillations, and K‐complexes progressively decrease with age, even before reaching advanced age.^87^, ^88^, ^89^ These changes impact sleep stability and cognitive function. Furthermore, ageing disrupts the coupling between ripples, sleep spindles, and delta oscillations, impairing neuronal communication and memory consolidation.^90^

Several sleep disorders, including sleep apnoea, insomnia, sleep‐disordered breathing, restless leg syndrome, and periodic limb movements, are associated with lifelong sleep alterations, though their prevalence may increase with age.

Ageing progressively affects sleep throughout life, not solely in old age. Sleep alterations, including circadian rhythm disruption, hormonal shifts, and changes in brainwave patterns, begin at earlier life stages and continue evolving over time. These disruptions contribute to daily functional challenges and hinder the ability to maintain cognitive and physical health. Furthermore, neurodegenerative diseases, which become more prevalent later in life, exacerbate sleep alterations, creating a complex interplay between ageing, sleep regulation, and neurodegeneration.

## 3 SLEEP IN NEURODEGENERATIVE DISEASES

### 3.1 Alzheimer's disease

Circadian rhythm disruption and sleep alterations are commonly observed in Alzheimer's disease patients, contributing to cognitive decline.^91^, ^92^, ^93^ Neurodegenerative changes, including hypothalamic neuronal loss and abnormal circadian rhythms, such as reduced core body temperature, are documented in patients and mouse models.^94^, ^95^ Transgenic 3xTg‐AD mice, which exhibit amyloid‐β (Aβ) and tau pathologies, display similar circadian disruptions, with decreased VIP‐positive cells suggesting an early predictive marker.^96^, ^97^ Melatonin levels are reduced in AD mouse models,^59^ while elevated cortisol levels in patients further support circadian rhythm impairment in early disease stages.^98^ The involvement of neurotransmitter systems in AD pathology is well documented. Serotonin plays a critical role in memory and learning via hippocampal modulation,^99^, ^100^ yet its levels are profoundly decreased in both patients and mouse models before Aβ accumulation, linking serotonin loss to exacerbated cognitive deficits.^101^, ^102^ Additionally, disruptions in orexinergic signalling have been implicated in AD progression. While cerebrospinal fluid (CSF) orexin levels are often found increased and positively correlated with Aβ burden,^103^, ^104^, ^105^ some studies report no significant difference between patients and controls,^106^ suggesting complex regulatory mechanisms. In AD mouse models, orexin knockout leads to reduced Aβ formation and improved sleep architecture.^107^ Furthermore, orexin dysregulation is associated with tau pathology, as increased orexin levels enhance tau and phosphorylated tau (P‐tau) accumulation, subsequently altering sleep macroarchitecture.^108^, ^109^ Beyond orexin, melanin‐concentrating hormone (MCH) neurons are implicated in AD pathology. MCH‐positive neurons are vulnerable in early disease stages, and increased CSF MCH correlates with P‐tau, total tau (T‐tau), disease severity, and dementia risk.^106^ Impaired MCH regulation may contribute to cognitive deficits, as disrupted REM sleep prevents effective memory consolidation.^110^, ^111^, ^112^ In AD models, progressive morphological alterations in MCH axons lead to aberrant neuronal activity and sleep alterations. Impairments in MCH trafficking and release further exacerbate pathology due to Aβ accumulation.^113^ Alterations in sleep architecture are strongly associated with AD progression. Increased sleep fragmentation accelerates cognitive impairment in the presence of Aβ and tau pathology.^114^, ^115^ Aβ further disrupts NREM3 sleep and memory consolidation.^116^ Mouse models exhibit fragmented sleep–wake cycles and shorter sleep bouts,^117^ while patients display altered sleep microarchitecture, particularly reduced sleep spindle density and slow oscillation activity.^118^ Sleep spindle deficits negatively correlate with P‐tau and T‐tau pathology, and disrupted sleep spindles‐slow oscillations coupling is linked to early Aβ burden, serving as an effective predictor of cognitive decline.^119^, ^120^, ^121^ Emerging evidence suggests that sleep spindles and slow oscillations may function as biomarkers for early AD diagnosis.^122^

### 3.2 Parkinson's disease

In Parkinson's disease, circadian rhythms are also disrupted. Melatonin levels were found to be decreased, and cortisol levels increased in patients.^98^, ^123^, ^124^ Serotonin levels are diminished in patients' CSF, which intrinsically correlates with the severity of motor and cognitive symptoms.^125^ Serotonin release was also affected, indicating a possible contribution to the worsening of non‐motor symptoms.^125^, ^126^, ^127^ In Parkinson's disease, there is a reduction in orexin levels, as evidenced by decreased concentrations in the CSF and a lower number of orexin‐positive neurons.^128^, ^129^, ^130^, ^131^, ^132^ The loss of orexin‐positive neurons in the lateral hypothalamus specifically disrupts the sleep–wake cycle of patients.^128^ This finding was confirmed in both a rat model of Parkinson's and a mouse model, where circadian rhythms and stability are impaired.^129^, ^133^ Alongside orexinergic loss, patients also had a loss of MCH‐positive neurons.^128^, ^132^ Patients experience excessive daytime sleepiness, as well as alterations in sleep patterns and increased occurrence of sleep apnoea.^134^ Several sleep disorders, including restless legs syndrome, REM behaviour disorder (RBD), and insomnia, have been associated with the pathophysiology of PD.^135^ Evidence suggests that RBD is a prodromal marker of PD, appearing 5 to 15 years ahead of Parkinson's onset.^136^, ^137^ Patients with RBD exhibit a lack of muscle atonia during REM sleep, physically acting out vivid, often unpleasant dreams accompanied by vocal sounds and sudden, usually violent, arm and leg movements.^138^, ^139^ The neuronal network responsible for muscle atonia comprises glutamatergic and glycinergic neurons located in the brainstem, and alpha‐synuclein deposits have been observed in the nuclei containing these neurons.^140^, ^141^, ^142^, ^143^, ^144^, ^145^ Patients show significant alterations in NREM3, which resulted in negative associations with cognitive functioning.^146^, ^147^ A mouse model also exhibits these circadian and homeostatic disruptions and sleep alterations, leading to a decline in locomotor activity.^148^ The disease also affects sleep microarchitecture, altering properties of sleep spindles such as their amplitude, which predicts cognitive deterioration and early decline of cortical regions.^149^, ^150^ Sleep spindles were found to be more profoundly impacted in patients who later developed Parkinson's dementia compared to patients without dementia and control individuals.^151^ The coupling between sleep spindles and slow oscillations can predict cognitive impairment in patients with Parkinson's disease. Sleep spindles are now considered potential biomarkers for predicting cognitive decline and disease progression.^152^, ^153^

### 3.3 Huntington's disease

Few studies have investigated circadian rhythms in Huntington's disease, particularly in patients. In Huntington's mouse models, mice exhibited complete loss of circadian rhythms, as well as disruption in NREM and REM sleep, with increased percentages of the two sleep stages.^154^, ^155^, ^156^ In patients, melatonin was found to be diminished compared to healthy individuals; conversely, cortisol levels were elevated.^157^, ^158^, ^159^ Melatonin biosynthesis is also disrupted in a mouse model.^160^ Unlike in Alzheimer's and Parkinson's, serotonin is only diminished in specific brain regions due to Huntington's aetiology in patients.^161^ Serotonin alterations were found in the R6/1 mouse model, which associates this loss with depression‐related behaviour.^162^ Several studies have shown a decrease in orexin levels and a loss of orexin‐positive neurons in individuals with Huntington's.^163^, ^164^, ^165^ Rodent models of Huntington's have also demonstrated a loss of orexin‐positive neurons,^166^, ^167^ and MCH‐positive neurons are lost in mouse models.^168^ Neuropathological investigations have revealed that patients suffer from hypothalamic atrophy and depletion of various hypothalamic neuropeptides, including orexin and oxytocin.^164^, ^165^, ^169^ Patients also show increased occurrence of insomnia and advanced sleep phase, and more rarely, restless legs syndrome or RBD. Huntington's patients also show reduced NREM 3 and REM sleep,^170^ increased wake after sleep onset and excessive daytime sleepiness.^171^ Q175 knock‐in mouse model has shown increased wakefulness and reduced NREM duration.^155^

### 3.4 Amyotrophic lateral sclerosis

Circadian rhythms have been poorly investigated in amyotrophic lateral sclerosis. Recent findings indicate circadian impairment and disruption of sleep–wake cycles. However, the circadian circuitry was devoid of pTDP‐43, an ALS hallmark.^172^ Circadian rhythms are also altered in ALS mouse models and have been linked to metabolic impairment and redox deficit.^173^ The mouse model displayed significant motor neuron loss, inflammation, and weight loss, resulting in an accelerated disease onset and progression.^174^ ALS patients were found to have significantly higher morning cortisol levels, leading to early wakefulness and disruption of their sleep–wake cycle.^175^ ALS is characterised by the degeneration of serotonergic neurons, resulting in reduced levels of serotonin.^176^, ^177^ The study found that the loss of serotonin exacerbates the progression of the disease and the severity of symptoms in mouse models, which was also replicated in humans.^178^ Despite hypothalamic atrophy prior to symptom onset, orexin levels remained unchanged in ALS patients.^179^, ^180^ The loss of MCH‐positive neurons early in the pathology is linked to weight loss and metabolic shifts in both patients and mouse models.^181^

Respiratory insufficiency developed by patients because of disease progression leads to increased sleep apnoea, which remains one of the most common sleep problems associated with amyotrophic lateral sclerosis. Sleep‐related disorders, such as hypoventilation, are also frequent due to the weakening of the diaphragm and paralysis of respiratory muscles.^182^, ^183^ Several studies suggest that individuals with ALS experience excessive daytime sleepiness, decreased total sleep time, insomnia, and increased sleep fragmentation, as well as RBD and restless legs syndrome.^184^, ^185^ Further investigation remains challenging due to the loss of innervation and early death caused by cardiorespiratory failure. In *TDP‐43*‐expressing *Drosophila*, severe sleep alterations are observed, characterised by reduced sleep duration and heightened sleep fragmentation. Yet, no circadian or homeostatic disruption was observed in this TDP‐43‐expressing fly model.^186^ However, in TDP‐43 knock‐out mouse models, clock gene expression is impaired, and locomotor activity is disturbed.^187^ SOD1^G93A^ mouse models have reduced core body temperature rhythm, indicating early central circadian dysfunction that progresses to peripheral thermogenic deficits as the disease advances, thus impacting sleep.^188^ In knock‐in rat model of FUS gene, REM sleep is deceased and wakefulness is increased at the begining of the active dark phase. These are associated with circadian deficits characterised by a dampened activity amplitude.^190^ Recent findings indicate that patients experience notable sleep alterations, including a reduction in restorative NREM3 sleep and increased wakefulness throughout the night. Similar findings were observed in presymptomatic gene carriers of ALS, indicating that sleep alterations may occur decades before the onset of the disease. Sleep alterations were also replicated in three different mouse models of amyotrophic lateral sclerosis, all of which exhibited increased wakefulness and disrupted NREM.^191^

### 3.5 Frontotemporal dementia

Sleep alterations have been the subject of extensive investigation regarding their disruptive impact on individuals with dementia yet have largely neglected frontotemporal dementia (FTD). Alterations in circadian rhythms have been observed in patients with FTD, with a tendency towards late wake‐up calls and later bedtime hours, suggestive of an evening chronotype.^192^ The findings were replicated in a Drosophila model of *C9ORF72*‐FTD, thereby providing evidence that circadian rhythms are indeed a hallmark of neurodegeneration.^193^ Another fly model of FTD for *CHMP2B* mutation also exhibited circadian defects, resulting in the disruption of circadian locomotor rhythms.^194^

It is noteworthy that patients with FTD display circadian rhythm impairments, as well as alterations in body temperature rhythm, that differ from those observed in Alzheimer's and control subjects.^195^ There is a paucity of studies that focus on circadian regulators. Several studies have attempted to assess the role of cortisol, and a causal link between cortisol levels and FTD has yet to be established.^196^ The most common diagnoses among FTD patients presenting with sleep‐related issues are sleep‐disordered breathing, excessive daytime sleepiness, and insomnia.^197^ A further decrease in plasmatic levels of orexin has been observed in patients with frontotemporal dementia in comparison to Alzheimer's and Parkinson's associated with mild cognitive impairments.^198^ This results in pronounced alterations to the sleep cycle, particularly in the NREM stage, accompanied by an increase in REM sleep.^199^, ^200^ A *FUS*‐FTD model replicated the neuronal loss observed in patients, including regions associated with orexinergic signalling. However, this loss was only detected in the locus coeruleus, where orexinergic neurons are not typically found.^201^ Additionally, elevated levels of tau were observed in the LHA, locus coeruleus and hippocampus of this mouse model. Despite the observation that CSF orexin levels are unchanged between patients and healthy individuals,^202^ disruptions in orexin signalling,^203^ such as altered receptor sensitivity or neuronal dysfunction, may still play a role in increased daytime sleepiness.^204^, ^205^, ^206^ In a *FUS*‐FTD rat model, impairment of circadian rhythms and alterations in sleep patterns precede cognitive decline.^207^

## 4 THERAPEUTIC APPROACHES TO SLEEP ALTERATIONS IN NEURODEGENERATIVE DISEASES

### 4.1 Melatonin

Melatonin has demonstrated neuroprotective effects across multiple neurodegenerative diseases. In Alzheimer's disease (AD) models, melatonin administration (10 mg/kg, intraperitoneally, daily for 4 weeks) reduced P‐tau and Aβ plaque accumulation, improving cognitive function and mitophagy.^208^ In Parkinson's patients, melatonin (3–5 mg orally, taken 30–60 min before bedtime) has shown efficacy in restoring circadian rhythms, improving sleep quality, and reducing sleep fragmentation.^209^, ^210^, ^211^, ^212^ It has also demonstrated neuroprotective effects in various Parkinson's models by reducing oxidative stress, inflammation, and apoptotic events and inhibiting synaptic dysfunctions as well as excitotoxicity.^213^, ^214^, ^215^, ^216^, ^217^, ^218^, ^219^ In Huntington's, melatonin (5 mg/kg, orally, daily for 3 weeks) restored circadian rhythms and locomotor abilities in a *Drosophila* model.^220^ The effect of melatonin has also been investigated in amyotrophic lateral sclerosis in both patients and mouse models. This indolamine (10 mg/kg, intraperitoneally, daily for 6 weeks) has been shown to have a neuroprotective effect on motor neurons, resulting in prolonged survival in mouse models and an antioxidant effect.^221^, ^222^, ^223^ It has been proposed as a potential candidate for altering the disease progression.^224^, ^225^ The efficacy of melatonin in frontotemporal dementia was evaluated in a clinical trial. However, in frontotemporal dementia (FTD) patients, melatonin (6 mg orally, nightly for 4 weeks) was found to exacerbate apathy, suggesting caution in its use.^226^

### 4.2 Melanin‐concentrating hormone

MCH supplementation has been explored in Alzheimer's disease models, where intracerebroventricular (ICV) administration of MCH (0.5 μg/day for 2 weeks) restored REM sleep and reduced abnormal hippocampal neuronal activity, improving memory retention.^113^, ^227^ In Parkinson's models, MCH supplementation (1 μg/day, ICV, for 3 weeks) exhibited neuroprotective effects on dopaminergic neurons, enhancing locomotor performance.^228^ The uptake of MCH has not previously been the subject of investigation in Huntington's patients or models. MCH supplementation (0.5 μg/day, ICV, for 2 weeks) was also performed in two ALS mouse models, but only partial rescue of sleep alterations was observed.^191^ MCH supplementation in these ALS models seemed to be able to prevent the neurodegeneration of motor neurons early in the disease course. A major limitation of current methods to target MCH is that MCH does not cross the blood–brain barrier and that its use is limited by its chronic intracerebroventricular delivery, while MCH neurons are mostly active during REM sleep. Better drugs targeting the MCH system would enable the activation of MCH at the appropriate period of the circadian rhythm.

### 4.3 Dual orexin receptor antagonist

Suvorexant, a dual orexin receptor antagonist (DORA), demonstrated a positive effect in Alzheimer's disease. Clinical studies indicate that suvorexant (10–20 mg, orally, taken 30 min before bedtime) reduces P‐tau and Aβ levels, improves sleep quality, and enhances memory retention.^129^, ^229^ Currently, no clinical trials have evaluated DORAs in Parkinson's disease, though preclinical studies suggest potential benefits. In Huntington's model, suvorexant (15 mg/kg, orally, daily for 4 weeks) rescued sleep alterations and improved cognitive function.^230^ A recent study from our group also unveiled that suvorexant administration (10 mg/kg, orally, acute) in mouse models of ALS effectively rescued sleep alterations at a presymptomatic stage.^191^

### 4.4 Other therapeutical approaches

Non‐pharmacological interventions have also been explored. In Alzheimer's patients, behavioural interventions, phototherapy (10,000 lux for 1 h in the morning), and continuous positive air pressure (CPAP) improved sleep quality, NREM2‐3 stability, and cognitive function.^231^, ^232^, ^233^, ^234^ In Huntington's disease, the administration of Ghrelin (0.3 mg/kg, subcutaneous injection, daily for 2 weeks) or Alprazolam (0.5 mg, orally, nightly for 4 weeks) reversed circadian rhythm disruptions, normalised sleep–wake cycles, and mitigated cognitive decline.^230^, ^235^, ^236^

## 5 DISCUSSION

The intricate relationship between sleep, lifelong ageing, and neurodegeneration underscores the critical role of sleep in maintaining neurological health. Sleep alterations, including reduced sleep duration, increased fragmentation, and shifts in chronotype, occur progressively with age, influencing both circadian rhythms and homeostatic sleep regulation. These disruptions, driven by hormonal imbalances, neuronal dysfunction, and alterations in sleep microarchitecture, have profound implications for cognitive function, emotional stability, and overall well‐being. Given that ageing itself is a continuous process that influences brain function, understanding how sleep disturbances contribute to neurodegenerative pathology is essential.

**FIGURE 1:**
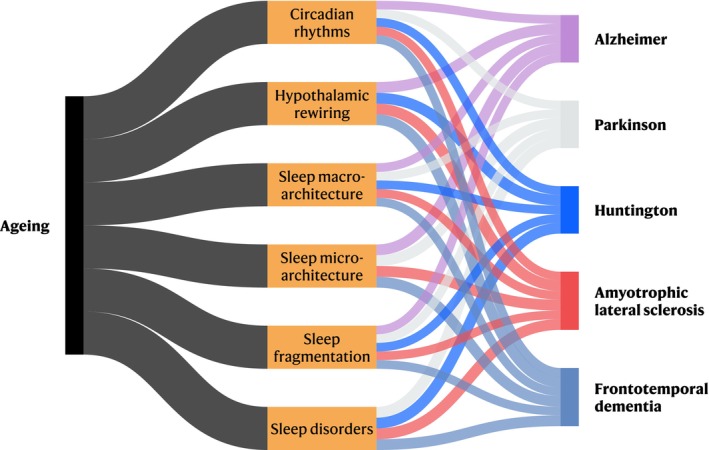
Interactions between sleep alterations, lifelong ageing and neurodegeneration. Sankey plot generated using the HoloViews package.^189^

In neurodegenerative diseases, sleep disruptions are often severe, multifaceted, and emerge early (Figure 1). Patients with Alzheimer's, Parkinson's, Huntington's, amyotrophic lateral sclerosis, and frontotemporal dementia experience progressive alterations in sleep architecture and circadian rhythms, frequently exhibiting sleep fragmentation, REM sleep abnormalities, and excessive daytime sleepiness. These disruptions correlate with the degeneration of key sleep‐regulating neural circuits, including the hypothalamic orexin and MCH systems. Furthermore, the bidirectional nature of these disruptions complicates intervention strategies, as neurodegeneration not only disrupts sleep, but inadequate sleep may also accelerate disease progression.

One of the most compelling aspects of recent research is the identification of sleep alterations as potential early biomarkers of neurodegenerative diseases. Changes in sleep microarchitecture, such as altered sleep spindle activity and reduced slow oscillations, have been associated with higher amyloid‐β and tau burden in Alzheimer's disease, and cognitive decline in Parkinson's disease. Similarly, sleep alterations in ALS and Huntington's disease precede clinical symptom onset, highlighting the need for early detection and intervention. Given that these alterations can emerge decades before overt neurological symptoms, incorporating sleep assessments into routine neurological screenings could provide valuable diagnostic insights.

Beyond diagnostic implications, the interplay between sleep and neurodegeneration may have direct mechanistic associations that further exacerbate disease progression. Sleep alterations impair glymphatic clearance, leading to reduced removal of neurotoxic proteins such as amyloid‐β and tau, which accelerates neuronal dysfunction.^237^, ^238^, ^239^ Additionally, fragmented sleep disrupts synaptic plasticity, memory consolidation, and neurotransmitter homeostasis, all of which are implicated in cognitive decline.^240^, ^241^, ^242^

Therapeutic strategies targeting sleep and circadian disruptions present promising opportunities for intervention (Table 1). Melatonin supplementation has shown efficacy in restoring circadian rhythm stability and improving sleep quality, particularly in Alzheimer's and Parkinson's disease models. Additionally, MCH supplementation has demonstrated neuroprotective effects, particularly in enhancing REM sleep and mitigating cognitive deficits. However, limitations such as the blood–brain barrier restrictions of MCH necessitate more precise therapeutic approaches. Dual orexin receptor antagonists, such as suvorexant, have shown promise in reducing amyloid‐β and tau accumulation in Alzheimer's disease, as well as ameliorating sleep alterations in Huntington's and ALS models. Beyond pharmacological interventions, non‐drug strategies, including light therapy, behavioural modifications, and continuous positive airway pressure, have demonstrated efficacy in stabilising sleep patterns and improving cognitive function in affected individuals.

**TABLE 1:** Therapeutic approaches targeting sleep alterations across neurodegenerative diseases.

	Alzheimer	Parkinson	Huntington	Amyotrophic lateral sclerosis	Frontotemporal dementia
Melatonin	Reduced P‐tau and Aβ plaque Improved mitophagy Stabilised circadian rhythms	Restored circadian rhythms, sleep quality Reduced oxidative stress, inflammation, excitotoxicity	Restored circadian rhythms and locomotor abilities	Neuroprotective effect on motor neurons Improved oxidative balance	Exacerbate apathy
Melanin‐concentrating hormone (MCH)	Restored REM sleep Reduced abnormal hippocampal neuronal activity Enhanced memory retention Lowered soluble Aβ	Neuroprotective effect on dopaminergic neurons Enhanced locomotor performance	—	Partial restoration of sleep alterations Early neuroprotection on motor neurons	—
Dual orexin receptor antagonists (DORAs)	Reduced P‐tau and Aβ plaque Improved sleep stability and memory retention	—	Restored sleep patterns and cognitive stability (preclinical evidence)	Restored sleep patterns and neuroprotective effects (preclinical evidence)	—
Light therapy	Improved NREM patterns Prevented excessive daylight sleepiness Enhanced cognitive functions	—	—	—	—
Ghrelin	—	—	Reversed atypical circadian rhythms Stabilised sleep–wake cycles Slowed down cognitive decline	—	—
Alprazolam	—	—	Reversed atypical circadian rhythms Stabilised sleep–wake cycles Slowed down cognitive decline	—	—

Further personalisation of sleep therapies is essential, as the heterogeneity of sleep alterations across neurodegenerative diseases necessitates targeted treatment strategies. For Parkinson's disease, interventions may focus on dopaminergic modulation and light therapy, whereas Alzheimer's patients may benefit from sleep spindles–enhancing therapies aimed at stabilising NREM3 sleep. The complexity of sleep regulation suggests that multimodal therapeutic strategies, combining behavioural, pharmacological, and chronotherapeutic interventions, may be required for optimal outcomes.

Despite these promising developments, significant challenges remain. Future research should clarify the precise mechanistic connections between sleep alterations and neurodegeneration and identify optimal intervention windows for therapeutic efficacy. Additionally, longitudinal studies tracking sleep biomarkers over decades could improve our understanding of how sleep changes predict neurodegenerative disease risk.

By advancing our understanding of the interplay between sleep, lifelong ageing, and neurodegeneration, we can develop more effective and targeted therapeutic interventions. The integration of behavioural, pharmacological, and chronotherapeutic strategies holds potential not only to enhance sleep quality but also to slow disease progression and improve the quality of life for affected individuals. Moving forward, prioritising sleep research in neurodegenerative disease models and clinical studies will be instrumental in uncovering novel treatment paradigms and refining early diagnostic methodologies.

## AUTHOR CONTRIBUTIONS

**Simon J. Guillot:** Conceptualization; visualization; writing – original draft; writing – review and editing. **Pierre‐Hervé Luppi:** Writing – review and editing; visualization; validation. **Luc Dupuis:** Conceptualization; supervision; writing – original draft; writing – review and editing; validation; funding acquisition. **Matei Bolborea:** Conceptualization; funding acquisition; writing – original draft; writing – review and editing; validation; supervision.

## CONFLICT OF INTEREST STATEMENT

The authors declare no conflicts of interest.

## PEER REVIEW

The peer review history for this article is available at https://www.webofscience.com/api/gateway/wos/peer-review/10.1111/jne.70085.

## Data Availability

Data sharing not applicable to this article as no datasets were generated or analysed during the current study.
